# Examination of the Tensile Strength of the Peroneus Longus Muscle

**DOI:** 10.7759/cureus.66683

**Published:** 2024-08-12

**Authors:** Hilal Yağar, Selim Cinaroglu, Fatih Çiçek, Faruk Ceranoğlu, Hüseyin Karadağ

**Affiliations:** 1 Department of Orthopedics and Traumatology, Niğde Ömer Halisdemir University, Niğde, TUR; 2 Department of Anatomy, Faculty of Medicine, Niğde Ömer Halisdemir University, Niğde, TUR; 3 Department of Anatomy, Niğde Ömer Halisdemir University, Niğde, TUR; 4 Department of Dentistry, İstanbul Gelişim University, İstanbul, TUR

**Keywords:** tensile force, anterior cruciate ligament, tendon graft, elongation, peroneus longus

## Abstract

Introduction: The peroneus longus tendon (PLT) is increasingly used as a tendon autograft in ligament and tendon reconstructions. The aim of this study is to evaluate the biomechanical properties of the PLT to assess its usability in frequently performed reconstructions.

Methods: Six fresh-frozen, below-knee cadavers with a mean age of 65 years, no previous surgical operation, and no history of chronic disease were used. PLTs were harvested, freed from muscle tissue, and prepared for tensile strength testing at a tensile force rate of 2 mm/min using a Shimadzu Autograph AG-IS 100 kN instrument (Shimadzu Corporation, Kyoto, Japan).

Results: The maximum tensile force varied between 600.7 N and 1131.313 N, with a median of 758.185 N. All tendons had diameters of 8 mm or more. The elongation at maximum force ranged from 9.0 mm to 16.0 mm, with a median of 14.0 mm.

Conclusion: According to this study, PLT is a viable choice for surgeries involving autograft reconstruction. However, further clinical studies are needed to confirm its efficacy in reconstructive surgeries.

## Introduction

The peroneus longus muscle is located on the lateral side of the lower leg and is posterior to the peroneus brevis muscle. It originates from the upper 2/3 of the upper outer surface of the fibula head, continues posteriorly to the lateral malleolus, and inserts at the first metatarsal and medial cuneiform bones [1]. The peroneus longus tendon (PLT) that terminates at the same place as the tibialis anterior muscle tendon suspends the foot and actively supports the foot arch [2]. PLT can be used as a source of tendon autograft in various surgeries for ligament and tendon reconstructions [3-5]. In anterior cruciate ligament (ACL) reconstruction, autografts (patellar tendon, quadriceps, peroneus longus, hamstring, etc.), allografts, and synthetic grafts can be utilized [6]. Recently, PLT has started to be frequently used as an autograft in ACL reconstruction, and it is considered to be thicker and stronger compared to hamstring tendons (HTs) [7]. It is also known that other tendons used in ACL reconstruction create recipient site morbidity around the knee [8]. It is important to understand the structural and mechanical properties of the tendons used in ligament reconstructions and to use tendon grafts that exhibit biomechanical behavior similar to or superior to that of the ligament being reconstructed [9]. Tensile loading tests are most commonly used to compare the biomechanical properties of tendons [10]. Tensile loading tests are calculated by measuring the uniaxial tensile loading of tendons that are rotationally stable [11]. The aim of this study is to evaluate PLT's tensile strength, which is used as a source of autograft for ligament and tendon reconstruction.

## Materials and methods

The Non-Interventional Ethics Committee of Niğde Ömer Halisdemir University provided the necessary ethical approval for this cadaveric study (decision number: 2024/xxx). The study utilized six fresh-frozen, below-knee cadavers with a mean age of 65 years (range: 62-88 years).

Tendon harvesting

**Figure 1:**
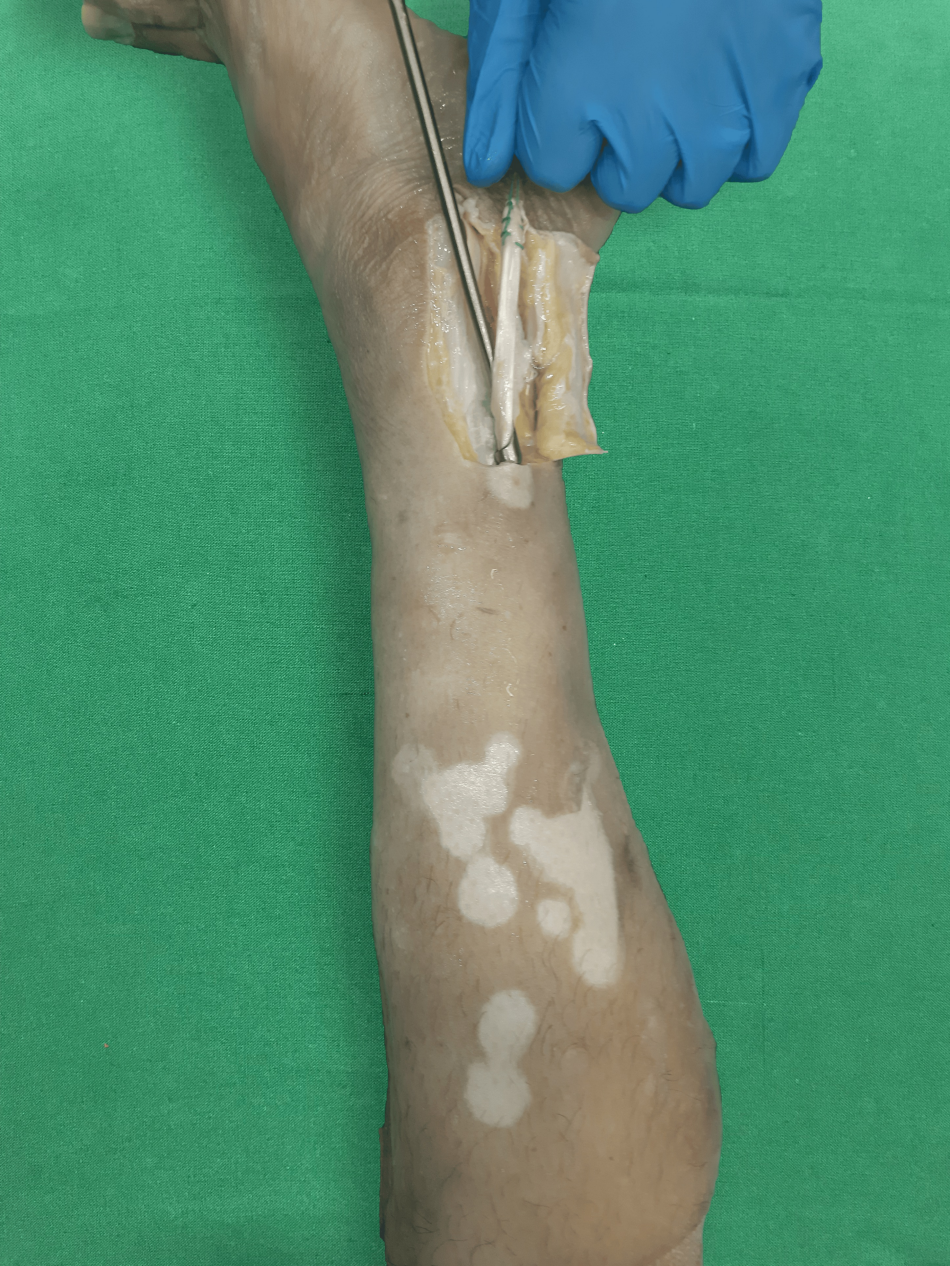
Harvesting the peroneus longus tendon using a tendon stripper.

The PLT was isolated and extracted from the posterior of the lateral malleolus after deviating an 8 cm portion of the skin flap laterally using a tendon stripper (Figure 1).

**Figure 2:**
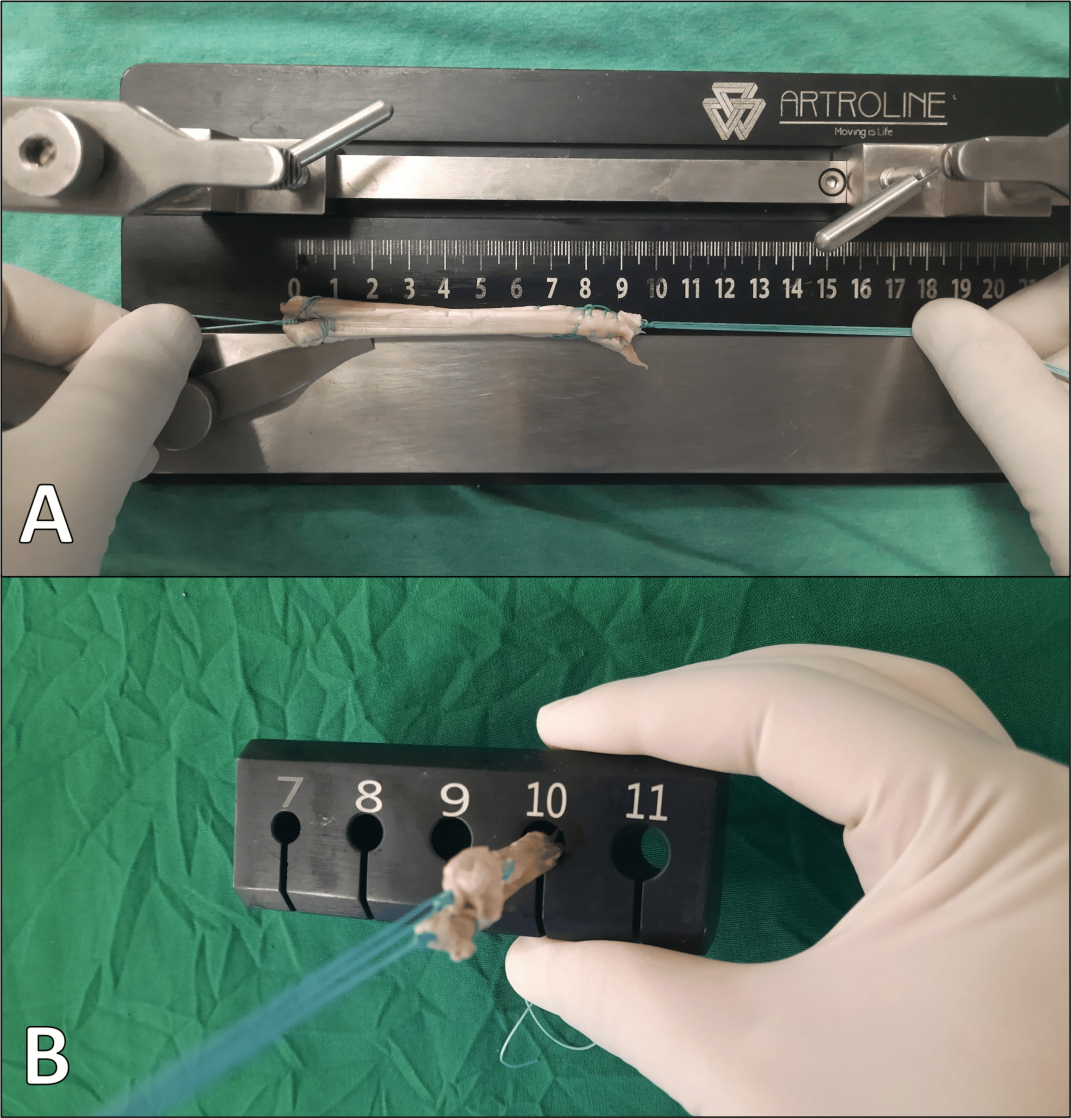
Measuring tendon diameter with a gauge. A: Measurement of tendon length. B: Measurement of tendon diameter.

It was then carefully cleared of the surrounding muscle tissue. Ethibond sutures were threaded through both ends using Krackow stitches, and then the tendon was doubled over. To mimic the method that the tendon is utilized in reconstructions, such as ACL reconstruction surgery where it is doubled over, the tendon is folded in half. The diameters of each tendon were measured using a gauge (Figure 2).

Tensile strength measurement

**Figure 3:**
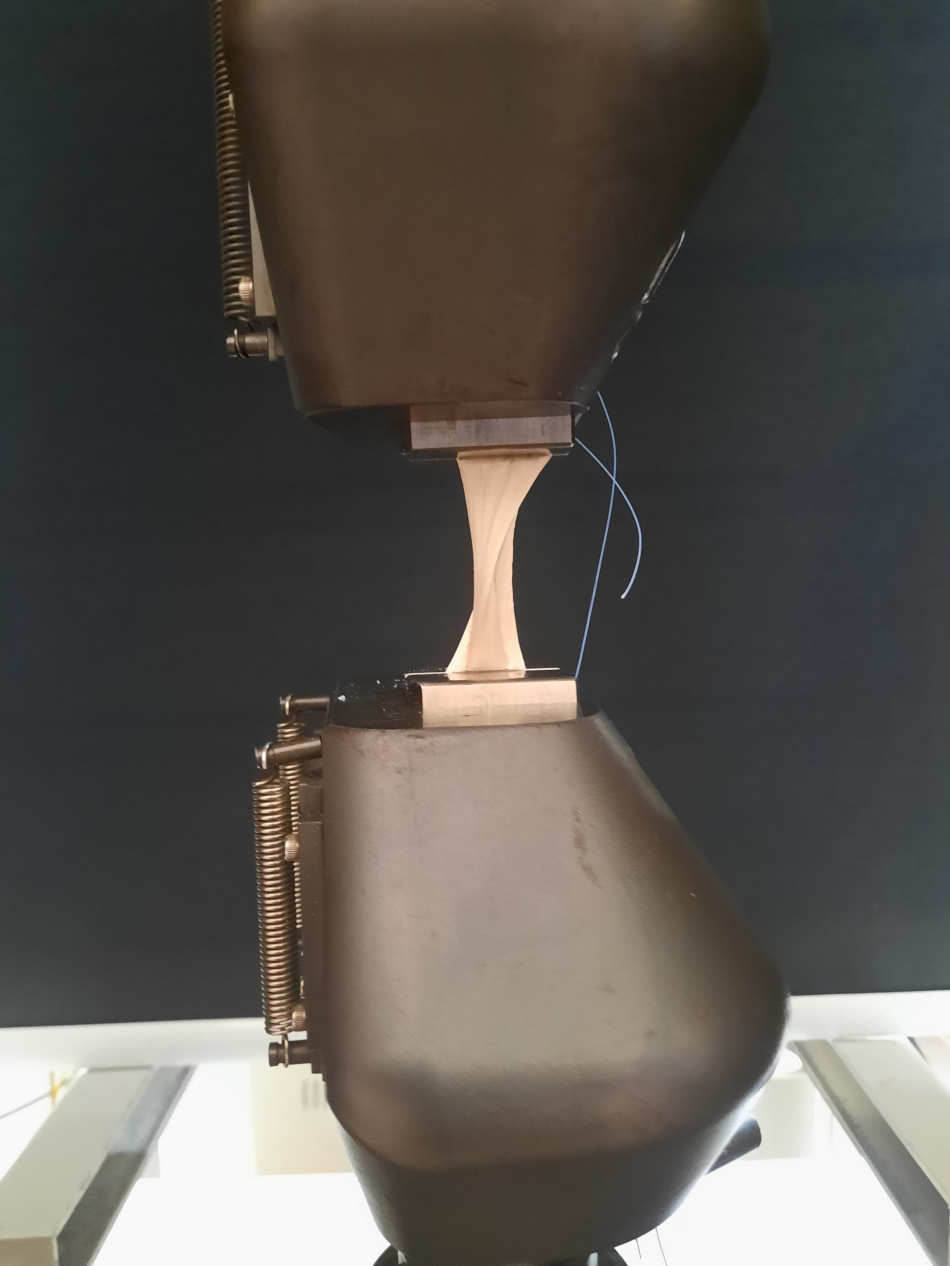
Measuring the tensile strength of tendons using the Shimadzu Autograph AG-IS 100 kN device.

The prepared tendons were tested using a Shimadzu Autograph AG-IS 100 kN device (Shimadzu Corporation, Kyoto, Japan), set to apply a tensile force at a rate of 2 mm/min. The grafts were secured to the device's grips along their long axis at the stitched areas using Ethibond sutures (Figure 3). For each sample, the tensile force values and the tendon elongation values up to the maximum were recorded separately.

Statistical analysis

Statistical analyses were conducted using SPSS software (IBM SPSS Statistics version 22, IBM Corp., Armonk, NY). For descriptive statistics, categorical data were presented as frequencies and percentages, while continuous data were presented as mean ± standard deviation or median (minimum-maximum).

## Results

**Figure 4:**
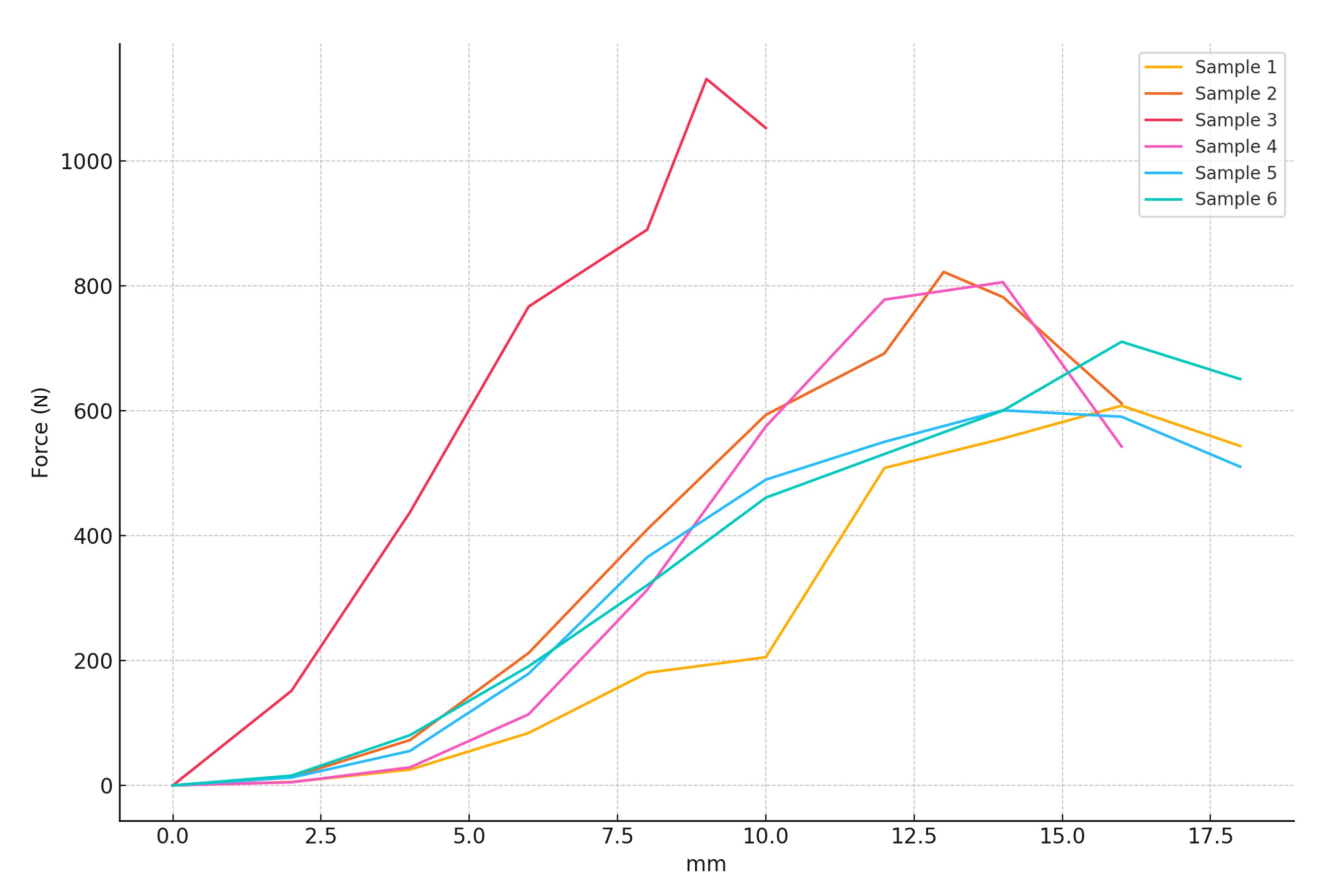
The relationship between tensile force (N) and elongation (mm) of samples.

The tensile force (N) and elongation (mm) values of the samples are shown in Figure 4. As seen in Figure 1, after the maximum force is applied, the force decreases because the tendon completes its oscillation or tension along the long axis. The sample with the least applied force is sample 1, with 608.156 N. The sample with the maximum applied force is sample 3, with 1131.31 N.

All tendons were found to have diameters of 8 mm or thicker. The samples and their respective tendon diameters are shown in Table 1.

**Table 1:** Diameters of samples after being folded in half.

Sample	Diameter (mm)
Sample 1	9
Sample 2	9
Sample 3	8
Sample 4	9
Sample 5	8
Sample 6	9

The maximum tensile force and the tendon elongation at maximum tensile force are shown in Table 2. The median tendon elongation at maximum force is 14.0 mm (min 9.0 - max 16.0). The median maximum tensile force is 758.185 N (min 600.7 - max 1131.313). Due to the working principle of the device, the tendon elongation's return to normal after the maximum force is applied could not be calculated.

**Table 2:** The maximum tensile forces and the tendon elongations at the maximum tensile force.

Sample	Max force (N)	Elongation (mm)
Sample 1	608.1563	16.0
Sample 2	822.31	13.0
Sample 3	1131.313	9.0
Sample 4	805.87	14.0
Sample 5	600.7	14.0
Sample 6	710.5	16.0

## Discussion

In this study, we demonstrated the tensile strength of PLT, one of the biomechanical properties of PLT, which is an important source of a tendon autograft. Phatama et al. found that the average tensile strength of the PLT was 223.50 N and that of the HT was 215.67 N in a cadaveric study. However, they did not find a statistically significant difference between the tensile strength of these two tendons (p > 0.05). They measured the tensile strength on unfolded tendons, finding an average of 223.5 N for the PLT, 215.67 N for the HT, 161.25 N for the patellar tendon, and 171.08 N for the quadriceps tendon [12]. In another biomechanical study where the tensile strength of the PLT was measured after being folded four times, the maximum tensile strength was found to be an average of 1170.4 ± 203 N [13]. In a biomechanical study using a hydraulic Servopulser tensile testing machine, unfolded PLT and HT were compared and an average tensile strength of 446.16 N for PLT and 405.88 N for HT was found. However, it was reported that no statistically significant difference was obtained (p > 0.05) [14]. Using the same device as in our study, Mert et al.'s cadaver study measured tensile strength and found that the maximum tensile strength in men was 807.7 for the HT, 405.18 for the patellar tendon, 315.13 for the quadriceps tendon, 868.52 for the Achilles tendon, and 70.83 for the ACL [15]. Markatos et al. reported that the mean tensile force of the ACL was 270 ± 1.72 N in their study [16]. Compared with our study, we see that the tensile strength of the peroneus longus (PL) tendon is higher than that of the ACL, quadriceps, and patellar tendon grafts and similar to that of the HT, which is consistent with the literature. Ashton et al. found that the tensile strength of the ACL was lower than the tensile strength of the PL in their study, which was statistically significant (p < 0.001) [17]. Agarwal et al. evaluated the surgical outcome of PL graft used in ACL reconstruction. They reported that PL graft can be a safe, feasible, and effective option for usual arthroscopic ACL reconstruction [18]. This shows that the PL tendon is sufficient in terms of tensile strength for ACL reconstruction. Reconstruction using the PLT is frequently applied in posterior cruciate ligament (PCL) ruptures, showing similar results to those achieved with the HT [19,20]. In previous studies, the maximum tensile strength of the PCL has been found to range between 739 N and 1627 N [21]. However, it is thought that the tensile strength of the PCL fibers might be higher due to their multi-axial orientation than the uniaxial tensile strength tests [22]. In our study, the maximum tensile strength of the PLT was found to be similar to that of human PCL, but further biomechanical studies are needed in this area. Another ligament where PLT reconstruction is applied is the coracoclavicular ligament [23]. In a biomechanical study, the tensile strength of the coracoclavicular ligament was found to be 724.9 ± 230.9 N [24], indicating that the coracoclavicular ligament's tensile strength is similar to that of the double-folded PLT in our study. In ACL reconstruction, having a smaller tendon graft diameter is associated with a higher risk of failure, with tendon thickness less than 8 mm doubling the failure rate [25]. In a study comparing the diameters of the HT and PLT, they found an average of 8.2 ± 0.8 for the HT and 8.8 ± 0.7 for the PLT, showing that the PLT graft diameter is larger [3]. In a prospective study of 50 patients comparing the diameters of the HT and PLT, the tendon diameter was found to be 7.43 ± 0.5 for the HT and 7.93 ± 0.52 for the PLT [26]. Similarly, in our study, we found that the diameter of all double-folded PLT specimens was 8 mm or more, consistent with the literature. Therefore, we believe that the PLT is a suitable alternative for ACL reconstruction in terms of tendon diameter. The main limitation of our study is the insufficient number of cadavers. In addition, since the cadavers were below-knee foot cadavers, tendon lengths could not be taken at their actual lengths and the measurements were not included in the study to avoid inaccuracies and bias. Another limitation of this study is that it could not be compared under similar conditions with other tendons used in reconstructive procedures. Moreover, many factors other than tensile strength play a role in tendon failure in reconstructive procedures.

## Conclusions

In conclusion, there are studies in the literature indicating that both mechanical and structural properties of the grafts should be evaluated in determining the most appropriate graft to be used in tendon reconstruction. Previous studies have reported that hamstring, patellar, and quadriceps tendons are commonly used in tendon and ligament reconstructions. In this study, the mechanical tensile strength of PLT was evaluated and it was revealed that PLT is a good alternative for surgeries requiring reconstruction with autograft. It is thought that clinical studies are needed to better evaluate the usability of PLT in these reconstruction applications.
